# Simulation and Optimization Study on the Ventilation Performance of High-Rise Buildings Inspired by the White Termite Mound Chamber Structure

**DOI:** 10.3390/biomimetics8080607

**Published:** 2023-12-14

**Authors:** Yangyang Wei, Zhiying Lin, Yihan Wang, Xinxia Wang

**Affiliations:** 1 Institute of Cultural Resources and Industries, Nanchang University, Nanchang 330031, China; 2 Architecture and Design College, Nanchang University, Nanchang 330031, China; 3 College of City Construction, Jiangxi Normal University, Nanchang 330022, China

**Keywords:** high-rise building, termite mound chamber, biomimetic architecture, ventilation environment, CFD simulation

## Abstract

High-rise buildings often use mechanical systems to assist ventilation to maintain the stability of their internal environments, and the energy consumption of mechanical ventilation poses a great challenge to urban environments and energy systems. The ventilation system of termite mounds with a combination of internal main and attached chambers is one of the classic examples of nature’s bionic approach to maintaining a stable internal ventilation environment for large-volume structures. In this study, based on the inspiration of the internal ventilation chamber structure of bionic termite mounds, we constructed seven high-rise building chamber ventilation models based on the chamber structure of termite mounds with main chambers, main chambers plus single-attached chambers (three types), and main chambers plus double-attached chambers (three types) under natural ventilation conditions, aiming at obtaining the optimal low-energy and high-efficiency chamber ventilation model for bionic termite mounds in high-rise buildings. (1) The wind speed and wind pressure of the high-rise building with the addition of the bionic termite mound chamber structure is higher than that of the traditional chamber-free high-rise building in the sample floors, the maximal difference of the wind speed between the two models is 0.05 m/s, and the maximal difference of the wind speed of the single building is 0.14 m/s, with the maximal difference of the wind speed of the single building being 0.14 m/s; and the natural ventilation environment can be satisfied by a high-rise building with a chamber. (2) After increasing the single-attached chamber structure of the bionic termite mound, the difference in wind speed of different floors is 0.15 m/s, which is 0.10 m/s higher than that of the high-rise building model with the main chamber only. (3) Under the bionic termite mound chamber high-rise building double-attached chamber model, the maximum difference in wind speed of each floor sampling point can reach 0.19 m/s, while the wind pressure cloud map shows a stable wind environment system. (4) Two attached chambers are added at A and B of the high-rise building to form the a4 model of the chamber of the high-rise building with a double-chamber bionic termite mound. According to the results, it can be seen that the model of the nine floor sampling points of the maximum wind speed difference has six places for the highest value, and the single building wind speed difference for the minimum value of 0.10 m/s. The study aims to optimize the connectivity and ventilation performance of high-rise buildings under natural ventilation conditions and to promote the green and sustainable design of high-rise buildings.

## 1. Introduction

Along with the increasing number and height of buildings in cities, high-rise buildings have a mutual influence on the overall climate of the city and the environmental quality of the buildings themselves [1]. The structural characteristics of high-rise buildings often require internal ventilation in addition to natural ventilation. Due to the structural characteristics of high-rise buildings, internal ventilation in addition to natural ventilation often also needs to rely on mechanical air-conditioning equipment to maintain the building’s internal wind environment, which is stable and comfortable, and relies on mechanical air-conditioning auxiliary equipment, which usually accounts for more than 40% of the energy consumption of high-rise buildings. The power consumption of mechanical air conditioning equipment usually accounts for more than 40% of the energy consumption of high-rise buildings [2]. Due to the large energy consumption base, the ecology and energy savings of high-rise buildings have been a hot research topic in the academic community, and how to effectively use the chamber space of high-rise buildings while reducing the energy consumption to maintain a stable indoor ventilation performance, in which the chamber of high-rise buildings in the realization of energy savings to improve the environment shows outstanding ecological prospects. From a bionic perspective, ventilation structures that mimic termite nest chambers may be a potential solution.

**Figure 1:**
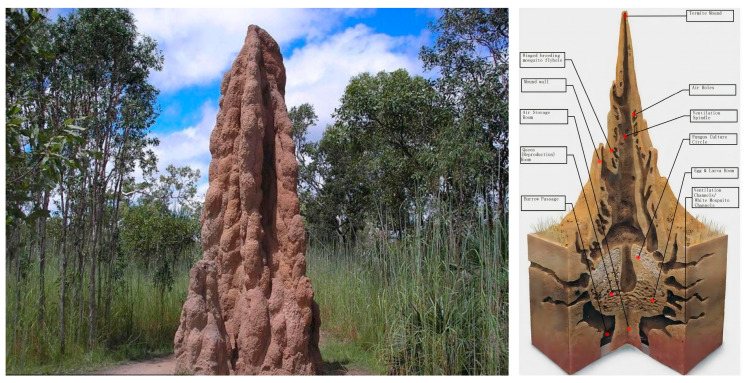
Anatomy of termite mound morphology and its internal structure.

Termites are swarming insects that build large mounds of earth to survive [3]. They build enormous mounds of earth to survive [4], the aboveground portion of which is called a termite mound, which often towers above the ground in the shape of a tower, column, or cone and can reach heights hundreds of times its own [5]. The termite mounds are shown in Figure 1. The structure of termite mounds is very similar to that of high-rise buildings, but the internal environment of termite mounds is extremely stable, and it has been shown that termites create a livable environment for themselves by changing the internal structure of termite mounds. The internal space of the termite mound shows a complex structure: the center of the interior consists of a main chamber and many compound chambers extending radially from the sides of the main chamber, which interact with each other to form a complete space for internal and external circulation [4]. This structure has been shown to be effective in utilizing natural ventilation to improve the microenvironment inside the termite mound to maintain a suitable temperature inside the nest at all times [6]. On the one hand, the specificity of the surface material of termite mound nests promotes the circulation and control of the temperature inside the termite mound chamber [7]. On the other hand, the ventilation and connectivity of the termite mound chamber can improve the gas flow and circulation in the chamber. Therefore, inspired by the chamber structure of termite mounds, the optimization of ventilation in high-rise buildings can be solved by the chamber structure of bionic termite mounds.

In the use of actual cases, a more typical one is the Eastgate Center in Zimbabwe designed by architect Mick Pearce, which is inspired by the design of termite mounds, a natural ecological wisdom. To provide the building with a good indoor temperature in summer and winter, borrowing the natural wisdom of termite nesting, through careful design and simulation calculations, from the spatial layout to the detailed design to the use of heat storage materials and façade texture, etc., a good passive ventilation and cooling system is constructed to control the temperature of the whole building, which finally provides the building with the same climate regulation function as the termite nests and greatly reduces the energy consumption of the building, and its passive ventilation and cooling system is also used in the building. The passive ventilation design of buildings has become one of the effective reference forms for the ventilation structure of bionic termite mound chamber high-rise buildings.

**Figure 2:**
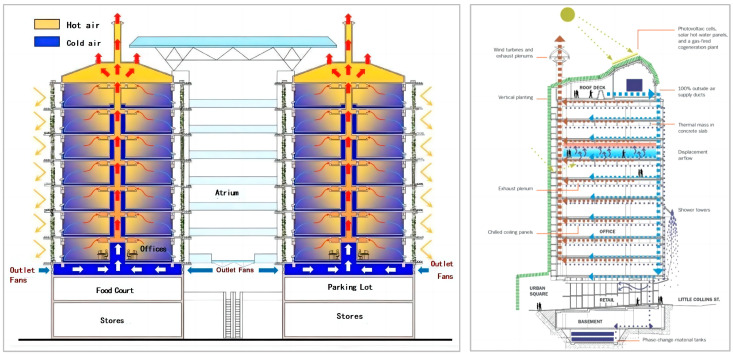
Schematic diagram of internal air ventilation in the Eastgate Center in Zimbabwe and the CH2 demonstration office building in Melbourne.

Similarly, Mick Pearce’s CH2 Melbourne demonstration office building is a model for future high-rise buildings, setting a world-class standard for sustainable design, with the Green Building Council of Australia rating the CH2 Melbourne demonstration office building as a six-star green building (six stars being the highest possible rating). The design of CH2 harmonizes the occupants with the surrounding environment. The atrium, which references a termite nest, is designed to be more functional than visual. Mick Pearce designed the building’s interior in reference to the workings of a termite colony, and CH2’s atrium features a precast corrugated concrete ceiling. The wavy space extends as far as possible to absorb the heat released by the occupants of the space below and to promote air flow. The building maximizes the use of internal ventilation improvements for temperature regulation and control, and the connectivity of the building is one of the key techniques used in the systematic solution to reduce the energy consumption of the temperature control, as shown in Figure 2.

With the development of computer technology, the use of computational fluid dynamics (CFD) technology to integrate natural ventilation design into building design and the optimization stage has become the mainstream of natural ventilation research. Lower simulation costs and more intuitive visualization of the results of CFD technology are significant features of [8]. Aghamolaei R utilized computational fluid dynamics (CFD) to develop a high-resolution yet computationally efficient program to couple radiative and convective fluxes in outdoor environments [9]. Wu P used CFD simulation to perform a comprehensive evaluation of atrium thermal environments, explaining the temperature distribution characteristics using heat-pressure theory and airflow theory [10]. Rabani M used computational fluid dynamics (CFD) to retrofit an ordinary office building in Oslo, Norway, with two different ventilation systems to improve user perception [11]. Chen L used computational fluid dynamics (CFD) simulation to reproduce the mean flow field around a single-building model and a two-building model to provide some insights for urban planners [12]. In summary, this study utilizes CFD to simulate the wind environment of high-rise buildings and to explore the wind environment of the bionic termite mound chamber structure of high-rise buildings.

**Figure 3:**
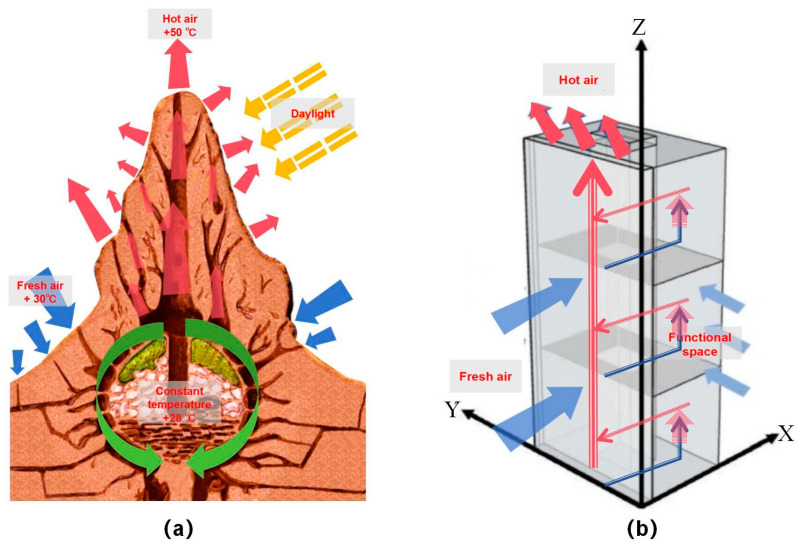
Bionic schematic: (**a**) Termite mound ventilation schematic. (**b**) Schematic diagram of bionic termite mound ventilation for a high-rise building.

It is not difficult to realize that for high-rise buildings, the height pattern is similar to the overall structure of a termite mound [13]. However, in the use of the actual bionic termite mound chamber building model, mechanical ventilation does not completely disappear. While minimizing the ventilation performance of high-rise buildings, maximizing the use of natural wind flow and imitating the use of termite mound chamber structures to improve the internal environment of high-rise buildings become the focus of this study. On the one hand, the ventilation performance of the interior of high-rise buildings can be optimized by relying on the flow of natural wind to maintain temperature. Especially in high-rise buildings with relatively large temperature differences between day and night, the question arises of how the internal ventilation performance of buildings, such as termite mounds, can be driven by internal air circulation with a relatively constant center temperature in response to temperature changes between day and night [14]. On the other hand, the connectivity between the floors of a high-rise building, i.e., the connectivity of the building, has become one of the key points of this study. On this basis, the study conducted the following CFD numerical simulation tests: the study conducted CFD simulation analysis on the original high-rise building model without chamber space and the high-rise building model with a main chamber space to verify that the main chamber structure can effectively improve the internal wind environment of the high-rise building. On the basis of the bionic termite mound high-rise building containing the main chamber, the attached chamber is added to strengthen the natural ventilation performance, and according to the different positions of the attached chamber, six kinds of high-rise building ventilation simulation schemes of the attached chamber structure are simulated under the main chamber high-rise building structure, in order to explore a more efficient scheme for high-rise buildings to use the chamber structure to improve the internal ventilation, as shown in Figure 3. According to Figure 3a, it can be seen that the hot air exhaust hole of the termite mound chamber is also in the upper part of the termite mound; thus, based on the judgment of air mobility performance, it can be seen that in the process of hot air exhausting from the building, the hot air is also in the upper part of the space in the established space. Therefore, according to Figure 3b, we add ventilation holes at the interface between the chamber and the indoor space, that is, the indoor space of each floor, so that the hot air inside the functional space can be brought together with the ventilation holes to the core ventilation chamber and then rise up to be discharged because of the air flow or the wind speed (wind pressure difference). It is worth noting that this study mainly utilizes the advantages of CFD simulation software from Heat and Momentum Limited (CHAM) PHOENICS (2019) to focus on the differences in wind speed and wind pressure of high-rise buildings in the bionic termite mound, while issues such as building temperature and air age have to be investigated in subsequent studies.

## 2. Materials and Methods

### 2.1. Object of Study: High-Rise Building Modeling

The CARRC [15] (Commonwealth Advisory Aeronautical Research Council) high-rise building model is a standard model proposed by the Federal Advisory Committee on Aeronautics in 1969, and its model dimensions are 45.72 m × 30.48 m × 182.88 m and rectangular. The model is often used to test the accuracy of wind tunnel test techniques and methods and is also used by many scholars as a benchmark model for research on the wind environment of high-rise buildings [16]. To simplify the simulation, the model was converted into a rectangular model. To simplify the difficulty of research simulation, the study sets the size of the base high-rise building model at 45 m × 30 m × 99 m. The length and width are set to facilitate the calculation of the percentage of the chamber to the volume of the model, and the height is set in accordance with the definition of the “Uniform Standard for the Design of Civil Buildings” (GB 50353-2019, China) [17], which stipulates that a building of more than 100 m is considered an ultrahigh-rise building.

**Figure 4:**
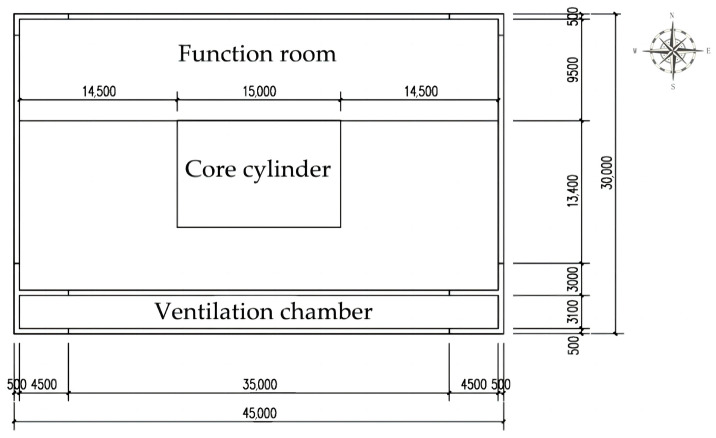
Standard high-rise building chamber plan diagram.

As shown in Figure 4, the model is set to have a floor height of 3 m, a total of 33 floors, and a centered core with dimensions of 15 m × 10 m. In setting the main chamber of the main chamber structure inside the same termite mound, according to the area share of the chamber structure inside the termite mound chamber [18], in this high-rise building model, a higher natural ventilation efficiency is achieved while preserving the functional space to the maximum extent possible [19]. Therefore, the model is set up with the main chamber accounting for 12% of the standard floor area, with a size of 45 m × 3.6 m. The openable window–wall ratio of the model is calculated as 10%, a ventilation window of 3 m × 0.5 m is opened on each floor of the east and west sides of the main chamber model, a ventilation opening of 35 m × 0.25 m is opened on the upper and lower sides of the south interface of the main chamber, and a ventilation opening of height 1 m × 0.25 m is opened at 1 m above ground level in the contact surface of the functional rooms and the chamber on each floor. Rectangular strip windows with a height of 1 m are also provided at 1 m above the ground on the contact surface between the functional rooms and the chamber.

**Figure 5:**
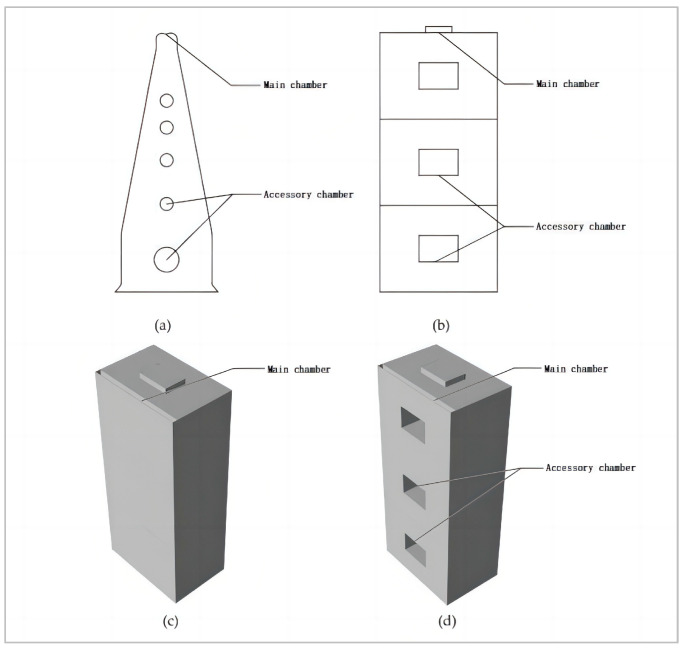
Schematic diagram of termite mound attachment chamber mimicry: (**a**) Sketch of termite mound attachment chamber. (**b**) Sketch of a high-rise building with an attached chamber (**c**) Model of a high-rise building with an attached chamber. (**d**) Sketch of the bionic termite mound attachment chamber model for a high-rise building.

It is worth noting that, according to the structural composition of the termite mound chamber, the main chamber and numerous attached chambers together promote the circulation and optimization of air within the termite mound chamber and are also key to achieving the connectivity of the internal structure of the termite mound chamber. To achieve a more accurate ventilation effect of the bionic termite mound high-rise building, according to the experimental setup, the study is based on the high-rise building containing the main chamber, and additional attached chambers are set to enhance the natural ventilation performance. To maximize the realization of the natural ventilation performance of the high-rise building model, the main chamber outside the interface sets up a larger area of the ventilation window to allow more natural air inflow; the main chamber is inside the interface because of the functional indoor space connected to the building, each floor is 1 m above the ground at the opening of a height of 1 m of the rectangular strip window, the formation of both is relatively independent and is set up to meet the linkage between the structure of the chamber. Based on this, the preliminary structural form of the high-rise building model, which relies on natural ventilation and contains the main chamber and the attached chamber, is constructed as shown in Figure 5.

**Figure 6:**
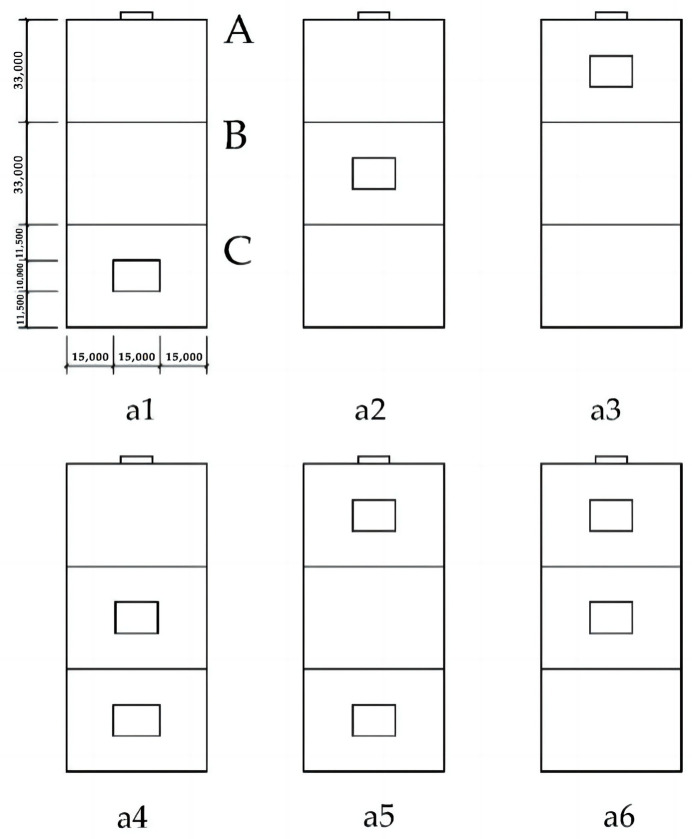
Six kinds of chamber measurement body diagrams.

To analyze the efficient scheme of the internal chamber structure of the bionic termite mound, after obtaining the standard model of the studied high-rise building with the main chamber, the morphological bionics of the attached chamber structure of the bionic termite mound are further carried out on the basis of the main chamber of the standard model. To reduce the energy consumption of the mechanical ventilation of the high-rise building model and to obtain a more efficient natural ventilation, the study proposes to set up a ventilation window with a larger area in the outer interface of the main chamber. However, according to Figure 3 and Figure 5, it can be seen that the number and size of the attached chamber openings of the termite mound chamber are not the same, the overall upper middle and lower parts will have more ventilation openings, and the purpose of opening different numbers of ventilation openings in different locations is to achieve the maximization of the ventilation performance of its interior. Based on this, analogous to the bionic high-rise building model of this study, under the relatively fixed setting of the internal interface, the different opening positions of the ventilation windows on the external surface will have different impacts on the wind environment of the whole building. To obtain more accurate data, the study divides the façade near the main chamber into three parts, A, B and C, increases the single-attached chamber and double-attached chamber in different positions to build six building models, and conducts CFD simulations individually. The reason for this setup is, on the one hand, to more accurately mimic the structural morphology of termite mound chambers and, on the other hand, to maximize the benefit of natural ventilation of the high-rise building model. The sketches of the six high-rise building ventilation attached chambers are shown in Figure 6.

### 2.2. Research Process

**Figure 7:**
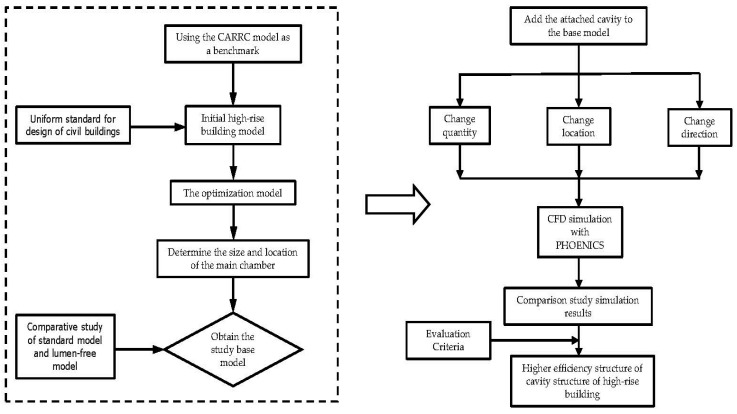
Research framework.

In the research process, the study first optimizes the CARRC international general high-rise building model to obtain the standard model for this simulation. Second, using the mathematical analysis method and the comparative research method, CFD simulation is carried out for the six types of altered attached chamber structural forms proposed by the standard bionic high-rise building model, and a comparative analysis of simulation effects is carried out. Finally, by combining the evaluation criteria set in the article, the model structural pattern with the optimal efficiency of natural ventilation in high-rise buildings using chamber structures is determined. The advantages of this research process include two main points. First, as with the termite mound chamber ventilation performance, natural ventilation is used to simulate the wind environment of the high-rise building model to minimize the energy consumption of the building. Second, through the comparison of six models, we explore the rationality of the distribution law of the accessory chambers in the same way as the termite mound ventilation structural pattern, i.e., we discover the optimal connectivity performance and the optimal model of the high-rise building model through the comparison of the models. The research framework is shown in Figure 7.

### 2.3. Evaluation Methodology and CFD Simulation Details

#### 2.3.1. Evaluation Methodology

The indoor wind environment evaluation standards in this study refer to the China Green Building Evaluation System, the Design Code for Heating, Ventilation, and Air Conditioning in Civil Buildings (GB 50736-2012, China) [20], and the standards established by some scholars’ research, which mainly include the following:

- Average indoor air velocity: the average air velocity of the cutoff plane at 1.0 m of the main working height of the human body in the functional rooms of each floor is selected as an evaluation index for analysis. Relevant research results show that when the indoor air velocity reaches 0.20~0.30 m/s, it can ensure a human physiological sensation in the comfortable range. The results of related research show that when the indoor air velocity reaches 0.20~0.30 m/s, it can make people feel comfortable [21];
- According to the code and previous research, the difference in wind pressure between the windward and leeward sides of a building exceeding 3 pa is used as an evaluation criterion for natural ventilation indoors [22];
- In this study, the CFD simulation data of the 11th standard floor, the 22nd standard floor, and the 33rd standard floor of the standard high-rise building model are used as the main evaluation basis for the average wind speed at the standard floor.

#### 2.3.2. CFD Simulation Details

Currently, the most common software used for CFD simulation are PHOENICS (2019), FLUENT (2019), STAR-CD (2019), Lady Bug (2019), etc. PHOENICS can be used to simulate the wind environment, thermal environment [23], solar radiation, etc. Many researchers use PHOENICS to simulate the wind environment of business complexes, office buildings, school buildings, residential clubs, etc. Yang Y used PHOENICS software to simulate the wind environment of the building complex in the central business district, which provides a reference theoretical basis and optimization basis for the development of relevant planning and design standards in the central district [24]. Liu J used PHOENICS software to simulate the thermal environment in an office with air-conditioned rooms in summer in a three-dimensional numerical simulation. Liu J utilized PHOENICS software to simulate the thermal environment in an air-conditioned office in summer [25]. Zhang L took a teaching building in Chengdu city as the research object, combined it with PHOENICS software to simulate the current situation of the indoor thermal environment in summer, and analyzed the impact of different optimization strategies on the indoor thermal environment of buildings in summer in hot summer and cold winter areas [26]. Chinglin M used CFD software PHOENICS to simulate the potential of natural ventilation and mechanical fan air conditioning for different scenarios under typical summer meteorological conditions in Sanya City and concluded that under the same indoor thermal comfort conditions, the wide application of mechanical fans in the tropics can reduce energy consumption [27]. Zhong J simulated the indoor thermal environment of the Wufu Clubhouse using PHOENICS software, and the simulation results showed that the ventilation could be improved by increasing the size of the air outlets (patio openings) and increasing the absolute difference of the surface wind pressure at the new openings [28]. In summary, PHOENICS software is suitable for simulation studies of indoor wind environments, and the calculation results are accurate and reliable. Therefore, PHOENICS was used for CFD simulation in this study. Its simulation settings are as follows:

Turbulence model equations: The Flair module of PHOENICS was chosen for simulation in this study, and there are three more general turbulence models [29].

(1) The *standard k-ε* is a simple industrial flow field and heat exchange simulation, with no large pressure gradient, separation, strong curvature flow, applicable to the initial parametric study, general building ventilation are applicable, and its formula as shown in Equations (1) and (2):

(1) $$\frac{\partial (\rho k)}{\partial t}+\frac{\partial \left(\rho ku_{i}\right)}{\partial x_{i}}=\frac{\partial }{x_{j}}\left(\alpha_{k}\eta_{eff}\frac{\partial k}{\partial x_{j}}\right)+G_{k}+\rho \varepsilon$$

(2) $$\frac{\partial (\rho \varepsilon )}{\partial t}+\frac{\partial \left(\rho \varepsilon u_{i}\right)}{\partial x_{i}}=\frac{\partial }{\partial x_{j}}\left(\alpha_{\varepsilon }\eta_{eff}\frac{\partial \varepsilon }{\partial x_{j}}\right)+\frac{C_{1s}^{*}\varepsilon }{k}G_{k}-C_{2s}\rho \frac{\varepsilon^{2}}{k}$$

where *k* is the turbulent kinetic energy.

*ε* is the turbulent dissipation rate.

(2) The *RNG k-ε* model is suitable for complex shear flows, including fast strain, moderate vortex flows, boundary layer separation, large angle stalls, room ventilation, and outdoor air flows, as shown in Equations (3) and (4):

(3) $$\frac{\partial }{\partial t}(\rho k)+\frac{\partial }{\partial x_{i}}\left(\rho ku_{i}\right)=\frac{\partial }{\partial x_{j}}\left(\alpha_{k}\mu_{eff}\frac{\partial k}{\partial x_{j}}\right)+G_{k}+G_{b}-\rho \varepsilon -Y_{M}+S_{k}$$

(4) $$\frac{\partial }{\partial t}(\rho \varepsilon )+\frac{\partial }{\partial x_{i}}\left(\rho \varepsilon u_{i}\right)=\frac{\partial }{\partial x_{j}}\left(\alpha_{\varepsilon }\mu_{eff}\frac{\partial \varepsilon }{\partial x_{j}}\right)+C_{1\varepsilon }\frac{\varepsilon }{k}\left(G_{k}+C_{3\varepsilon }G_{b}\right)-C_{2e}\rho \frac{\varepsilon^{2}}{k}-R_{\varepsilon }+S_{\varepsilon }$$

where $G_{K}$ denotes the generation of turbulent kinetic energy due to the mean velocity gradient.

$G_{b}$ indicates the turbulent kinetic energy generated by buoyancy.

(3) The *Realizable k-ε* model is suitable for extremely complex flow condition model simulation calculations, such as rotating flow, strong counterpressure gradient of the boundary layer flow, flow separation, and secondary flow, and its equations are shown in Equations (5) and (6):

(5) $$\frac{\partial }{\partial t}(\rho k)+\frac{\partial }{\partial x_{j}}\left(\rho ku_{j}\right)=\frac{\partial }{\partial x_{j}}\left[\left(\mu +\frac{\mu_{t}}{\sigma_{k}}\right)\frac{\partial_{k}}{\partial x_{j}}\right]+P_{k}+P_{b}-\rho \varepsilon -Y_{M}+S_{k}$$

(6) $$\frac{\partial }{\partial t}(\rho \varepsilon )+\frac{\partial }{\partial x_{j}}\left(\rho \varepsilon u_{j}\right)=\frac{\partial }{\partial x_{j}}\left[\left(\mu +\frac{\mu_{t}}{\sigma_{\varepsilon }}\right)\frac{\partial_{\varepsilon }}{\partial x_{j}}\right]+\rho C_{1}S_{\varepsilon }-\rho C_{2}\frac{\varepsilon^{2}}{k+\sqrt{\nu \varepsilon }}+C_{1_{e}}\frac{\varepsilon }{k}C_{3_{\varepsilon }}P_{b}+S_{\varepsilon }$$

where ρ is the density of air.

$P_{k}$ represents the turbulent kinetic energy due to the mean velocity gradient.

$P_{b}$ is the turbulent viscosity.

Combined with the characteristics of the object simulated in this study and the amount of data studied, the simulation of the working conditions in the process is not a large pressure gradient, separation, or strong curvature of the flow, so there is no need to choose a more complex simulation of the working conditions of the computational model. In the focus of the study, the experiment focuses on the analysis of bionic high-rise building ventilation efficiency and the building form for the monolithic high-rise building form structure, focusing on the simulation of the monolithic high-rise building wind environment characteristics of the interface of the outside of the building, and therefore does not involve the details of the indoor air convection. Therefore, to increase the simulation of the efficiency and effectiveness of the simulation, the simulation in the work of the selection of a more direct and efficient standard *k-ε* turbulence model for the calculation of the outdoor flow field of a single high-rise building. To increase the efficiency and effectiveness of the simulation, the *standard k-ε* turbulence model is chosen for this simulation [30].

Grid settings: The grid settings are based on the software’s own modules and the Green Building Evaluation Standard (GB/T 50378-2019, China) [31]. To improve the simulation accuracy and reduce the number of grid segments, the calculation area is divided into the center area (fine grid segments) and the edge area (coarse grid segments). According to the *standard k-ε* turbulence modeling operation of the fitness, the length and width of the boundary are 4 times the model height H [32]. The height of the boundary is also 4 times the maximum house height H degree of the model. The simulated object is located in the center of the region. This is iterated until the computational error converges [33]. Due to the different requirements for computational accuracy in different regions of the simulation, different regions of the mesh were encrypted.

**Figure 8:**
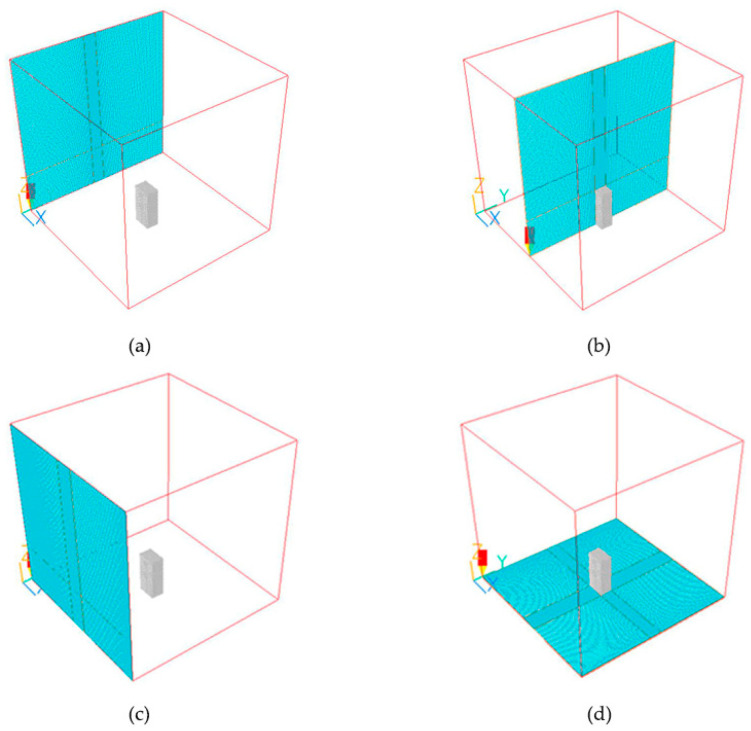
PHOENICS mesh settings: (**a**) Schematic of the mesh on one side of the x-axis. (**b**) Schematic of the mesh in the middle of the x-axis. (**c**) Schematic of the right side of the y-axis mesh. (**d**) Diagram of the z-axis mesh.

The grid size is set as follows: In the X and Y axis planes, the grid of the research object area located in the center is set as a fine segment with a size of 3 m × 3 m; the edge area without a model is a rough segment with a size of 5 m × 5 m. The stretch ratio at the transition of the coarse and fine grid areas in the x- and y-axes is 1.2, and both of them are tightened in the direction close to the building. For the z-axis in the vertical direction, the grids of the region are set as follows: a grid line of 0.5 m is set from the ground to the height of the building at 1.5, a grid line of 2 m is set from 1.5 m to the top of the building, and a grid line of 3 m is set from the top of the building to the boundary of the simulation range at a distance of 3 m. It is worth noting that in this simulation, different grid sizes are set for the simulation according to different regional model location characteristics or the degree of roughness and fineness of the model. Different network data do not affect the unity of the simulation environment, and setting different grid parameters can not only ensure the accuracy of the simulation calculation but also reduce the time spent on simulation and improve the efficiency of simulation. After calculation, the total number of grids under the standard model is 2.475 million, and the overall grid is shown in Figure 8.

Other working condition settings: According to the Design Code for Heating, Ventilation, and Air Conditioning of Civil Buildings (GB 50736-2012, China) [20] and the on-site wind conditions in Nanchang City (115°27′–116°35′ E longitude, 28°09′–29°11′ N latitude), a summer average wind speed of 3.1 m/s (WSW) was selected as the incoming boundary condition. The number of iterations is 1000. Nanchang city is an urban area with many house buildings, and the ground roughness index is selected to be 0.28.

## 3. Results

In this study of the internal chamber structure of the bionic termite mound body in high-rise buildings, a standard test model with a main chamber was set up with three bionic termite mound single-attachment chamber models and three bionic termite mound double-attachment chamber models. The experimental results contain three main parts:

The first part is a comparative CFD simulation study between the base chamber-less high-rise building model and a standard test model with a main chamber;

The second part adds a single-attached chamber at different locations near the side of the main chamber and compares and studies the effects of the different locations of the three different attached chambers, A, B, and C, as divided in Figure 6, on the ventilation performance of the high-rise building model;

The third part is a comparative study of the effect of three double-attached chamber forms on the ventilation of high-rise buildings.

### 3.1. Comparative Study of High-Rise Building Models without Chambers and High-Rise Building Models with Main Chambers

**Figure 9:**
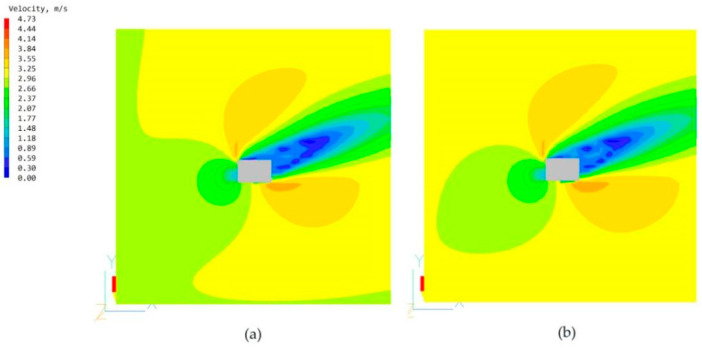
Wind speed maps at the pedestrian walkway (1.5 m) without and with the chamber model: (**a**) Wind speed maps with the chamber model. (**b**) Wind speed map with the main chamber model.

**Figure 10:**
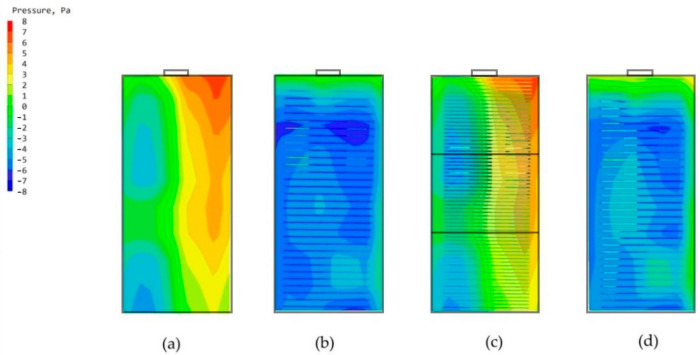
Windward and leeward pressure maps: (**a**) Windward side of the modeled body without chambers. (**b**) Leeward side of the model without a chamber. (**c**) Windward side of the model with a chamber. (**d**) Leeward side of the model with a chamber.

**Figure 11:**
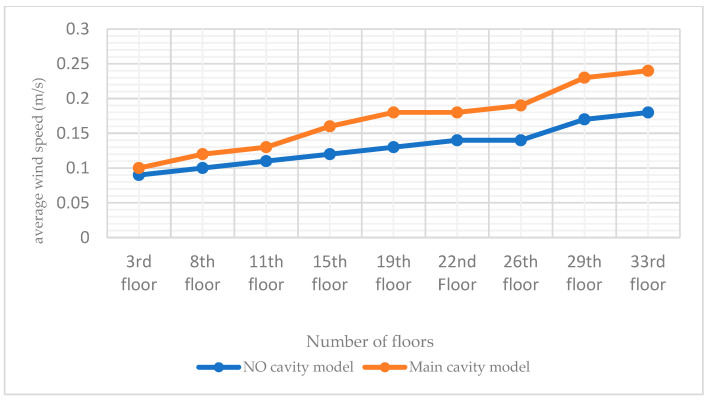
Plot of average wind speeds in selected standard layers of the model without and with chambers. The data in the figure are derived from CFD simulation results.

The high-rise building model with no chamber and the building model with only the main chamber are imported into the PHOENICS software for CFD wind environment simulation. After simulation, the simulated wind speeds of the two models at the pedestrian walkway (1.5 m) are shown in Figure 9; the windward-tunnel and leeward pressure maps are shown in Figure 10; the average wind speeds of the two models at some of the standard floors (including the 11th, 22nd, and 33rd floors) are shown in Figure 11.

### 3.2. Study of Single-Attached Chambers at Different Locations

**Figure 12:**
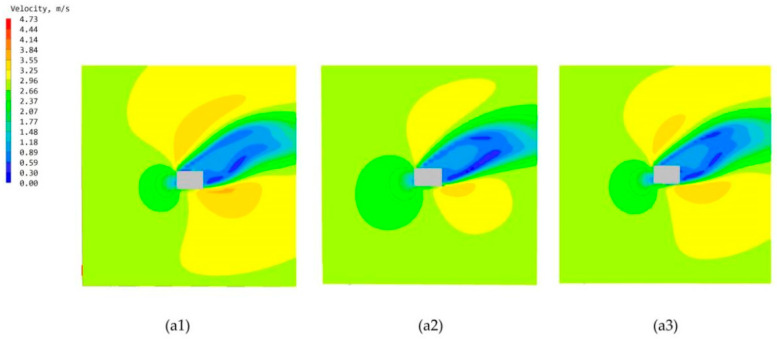
Wind speed maps of three single-attached chamber models at the pedestrian (1.5 m): (**a1**) Model a1 wind speed map. (**a2**) Model a2 wind speed map. (**a3**) Model a3 wind speed map.

**Figure 13:**
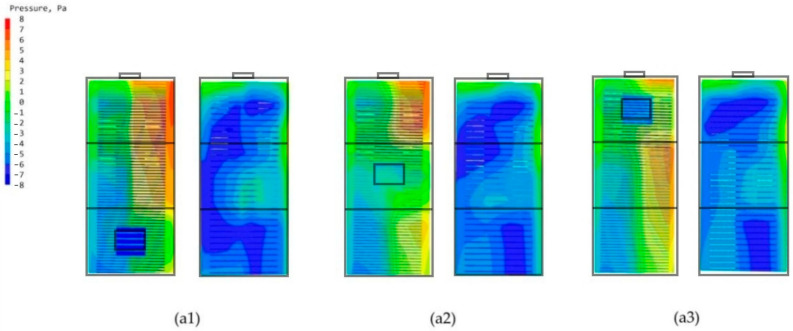
Windward and leeward pressure clouds: (**a1**) Model a1 windward and leeward surfaces. (**a2**) Model a2 windward and leeward surfaces. (**a3**) Model a3 windward and leeward.

**Figure 14:**
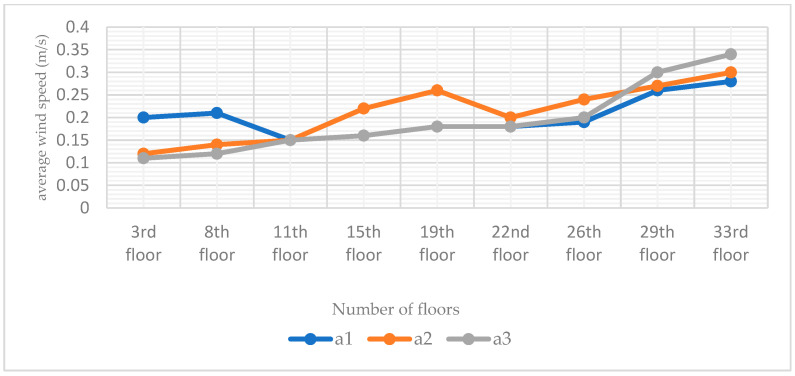
Line plots of mean wind speeds in selected standard layers for the three single-attached chamber models (a1, a2, and a3). The data in the figure are derived from CFD simulation results.

The standard high-rise building model is only set up with the main chamber, and the most important reason that can constitute the internal and external circulation in the structure of the termite mound is the joint structure of its main chamber and attached chambers. According to the structural composition of the termite mound chamber, the main chamber and numerous attached chambers jointly promote the circulation and optimization of the air within the chamber of the mound, and they are also key to achieving the connectivity of the internal structure of the chamber of the termite mound to enhance the biomimetic characteristics of the high-rise building model. To enhance the bionic characteristics of the high-rise building model and improve the effectiveness of the simulation, in this study, a single-attached chamber is first set up on the side close to the main chamber, and three different positions (evenly divided) are set up on the external surfaces A, B, and C of the high-rise building model (with the main chamber), which results in the formation of the three bionic high-rise building model morphologies of a1, a2, and a3, as shown in Figure 6. To investigate the influence of the attached chambers at different locations on the ventilation performance of the high-rise building, the study conducted CFD simulations of these three models. After the simulation operation, the simulated cloud diagrams of wind speed at the pedestrian place (1.5 m) of the three are obtained as shown in Figure 12; the cloud diagrams of pressure on the windward and leeward surfaces of the three are shown in Figure 13; the average wind speed of the three partially standardized floors is shown in Figure 14.

### 3.3. Study of Double-Attached Chambers in Different Arrangements

**Figure 15:**
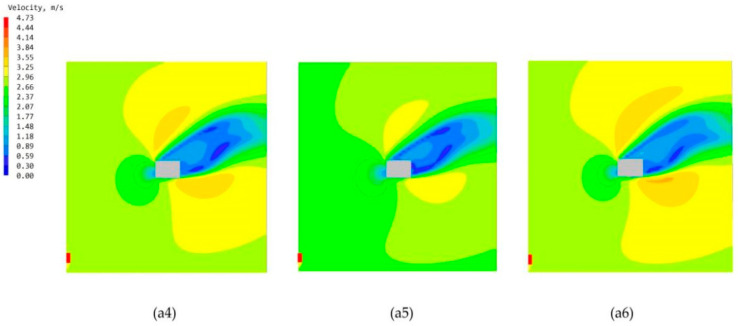
Wind speed maps of three double-attached chamber models at the pedestrian walkway (1.5 m): (**a4**) Model a4 wind speed map. (**a5**) Model a5 wind speed map. (**a6**) Model a6 wind speed cloud.

**Figure 16:**
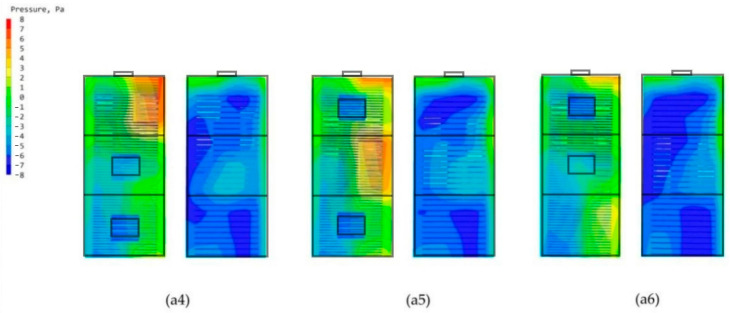
Windward and leeward pressure clouds: (**a4**) Model a4 windward and leeward. (**a5**) Model a5 windward and leeward surfaces. (**a6**) Model a6 windward and leeward surfaces.

**Figure 17:**
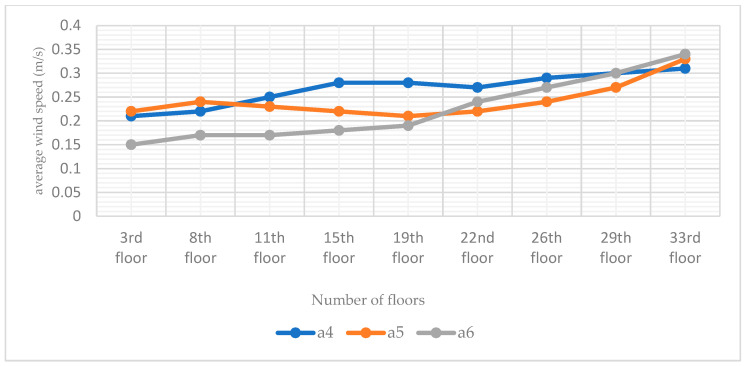
Line plots of mean wind speeds in selected standard layers for the three double-attached chamber models (a4, a5, and a6). The data in the figure are derived from CFD simulation results.

From the chamber structure of the termite mound, it can be found that the attachment chamber system is composed of multiple attachment chambers. The distribution number of chambers in different locations is not the same, and the different number of different areas naturally leads to different open areas of the attached chambers. Therefore, to study the wind environment characteristics of the bionic termite mound chamber structure high-rise building model under natural ventilation conditions, this study, after studying the performance of different individually attached chambers, sets up a double-chamber structure on the side close to the main chamber, and the combination of the two attached chambers is distributed in different locations, which forms three structural forms of a4, a5, and a6, as demonstrated in Figure 6. Such a setup, on the one hand, improves the authenticity of the bionic and, on the other hand, can also maximize the exploration of the optimal ventilation performance of the high-rise building model under natural ventilation conditions. After the simulation operation, Figure 15 shows the three forms of high-rise building pedestrian office [34]. The simulated cloud diagrams of wind speed, and the pressure cloud diagrams of the windward and leeward sides of the three are shown in Figure 16; and the average wind speed data of CFD simulations of some standard floors of high-rise buildings with different double-attachment chambers modes are shown in Figure 17.

## 4. Discussion

This study builds a high-rise building model applicable to the situation of this study from the CARRC high-rise building model and combines it with the current Chinese codes. From the internal structure of termite mound chambers, we know that one of the main chambers that connects the inside and outside of the structure is the main factor that ensures its performance. Therefore, we added the main chamber in the standard building model, which needs to consider the constraints of the use of high-rise buildings and the use of the chamber to optimize the environmental quality of the functional space but cannot affect the functional use of the intrinsic performance, so the location of the main chamber of the model for this study is in the high-rise building model of the long side of the side, as shown in Figure 3 of its scale size according to energy-saving specifications. To confirm the role of the main chamber, CFD simulations are performed by PHOENICS software on the model with and without the main chamber. For the comparability of the study, it is uniformly stipulated that the CFD simulation conditions of this study are based on the summer ventilation conditions in Nanchang City. From Figure 9, the wind speed maps at the height of the pedestrian (1.5 m) of the two models show that the increase in the chamber has a negligible effect on the environment around the building itself, which is the basis for us to explore the effect of the chamber structure on the high-rise building. By comparing the windward and leeward sides of the two models in Figure 10, it can be seen that more than 80% of the area of the windward and leeward sides of the wind pressure difference between the front and back is more than 3 Pa, and the building’s indoors are able to utilize the natural ventilation effect. As shown in Figure 11, by comparing the average wind speed of some standard floors of the two, it can be seen that the wind speed of the floor of the high-rise building model from the 11th floor of the 0.11 m/s to the 22nd floor of the 0.14 m/s to the 33rd floor of the 0.18 m/s is greater with the increase in height, showing a rising trend. After the increase in the main chamber structure, the overall wind speed of the standard floor of the high-rise building as a whole is increased, such as the average wind speed of the 8th floor increasing from 0.1 m/s to 0.12 m/s. In addition, for some of the lower floors (3rd and 8th floors), after the increase in the main chamber model, the average wind speed tends to be closer to the midpoint of the human body’s appropriate wind speed, as shown in Table 1.

From the structure of termite mounds in addition to the main chamber structure, there are also many attached chambers, whose function is to assist the main chamber in ensuring the environment in the structure of termite mounds maintains balance and stability. To further explore the chamber structure of the bionic termite mound, this study proposes six forms of attached chambers, as shown in Figure 6, to demonstrate the effect of attached chambers on high-rise buildings. It is divided into three parts, A, B, and C, on the side close to the main chamber. The effect of a single chamber on the high-rise building is first explored by adding a single-attached chamber to each of the three sections (a1, a2, and a3), with the openings of the added chamber having the same area as the cross-sectional area of the main chamber. After the addition of a single-attached chamber, observing the wind speed cloud diagrams at the pedestrian walkway of the three in Figure 12, it can be seen that the attached chamber does not have a significant impact on the overall surrounding wind environment of the high-rise building. As shown in Figure 13, the windward and leeward surfaces of the building surface pressure maps and the windward and leeward surfaces of the three models reach the indoor pressure that can be utilized for natural ventilation, and the part of the model that has an attached chamber has a smaller difference in wind pressure than that of the part that does not have an attached chamber, e.g., the front and rear wind pressure difference in part C of the model a1 is smaller than that in parts A and B. The wind pressure difference in part C of the model a1 is larger than that in part B of the model a1. From the corresponding three models of the standard floor of the average wind speed value of Figure 14, the standard floor wind speed of the three are in line with the law of gradual increase in the trend of the standard floor, in which the same floor in the standard floor near the attached chamber of the floor of the wind speed is higher than away from the attached chamber in the case of the wind speed, such as the 8th floor of the a1 model (the attached chamber is near the 8th floor) of the wind speed of 0.21 m/s but in the a3 (the attached chamber is away from the 8th floor) model has become 0.12 m/s, the influence of the attached chamber is gradually weakened with the distance, as shown in Table 2.

By observing the distribution of ventilation holes in the chamber structure of termite mounds, it can be seen that different areas distribute different numbers of ventilation holes, which leads to different effects of different areas on the overall ventilation performance of termite mounds. Based on this, the study proposes to maximize the area of the external interface of the ventilated chamber under natural ventilation conditions. Therefore, after the simulation of the single-chamber model, this study conducts CFD simulations of three high-rise building models in the form of dual chambers (a4, a5, and a6), and it is clear from the wind speed clouds at the pedestrian walkway in Figure 15 that the three dual-attached chamber structures do not significantly change the environment around the building. As shown in Figure 16, in the mode of the double-attached chamber, the model also appears in the part of the windward and leeward surfaces near the chamber of the pressure difference between the front and rear to be smaller than the case away from the chamber. Observing Figure 17, it can be seen that the mean wind speed of the standard layer of the double-attached chamber structure shows the same increasing pattern as that of the single chamber, but it is worth noting that the wind speed of the standard layer in the part of the double-attached chamber model without an attached chamber is higher than that of the same in the case of the single-attached chamber model. As shown in Table 2, the average wind speed of the 33rd floor of model a1 is 0.28 m/s, while that of the 33rd floor of model a4 is 0.31 m/s. On the other hand, since part A is at the highest position of the overall model, the original wind speed of its base model is higher than that of the other two parts, and setting up the attached chamber in part A will lead to high wind speeds in the interior of part A, e.g., the wind speed of the 33rd floor of the model a6 reaches 0.34 m/s. This model is consistent with the basic properties of air flow in a termite mound chamber structure, where hot air is pooled into the main chamber through air holes and exhausted upward, while the a4 bionic high-rise building model is also consistent because of the air entry from the bottom two attached chambers, which realizes the hot and cold air mobility replacement under the condition of natural ventilation. Therefore, care should be taken to avoid floors where the average wind speed has reached the standard in the original case when setting up the attached chamber to assist the main chamber to avoid the phenomenon of excessive wind speed.

## 5. Conclusions

High-rise buildings often add intrusive mechanical systems to the building to ensure that the environment of the high-rise building builds a more stable indoor environment and increases the comfort of users [35]. The energy consumption of these intrusive mechanical systems accounts for the total building energy consumption. The energy consumption of this intrusive machinery occupies more than 40% of the energy consumption part of the whole building, and this approach poses a great challenge to the urban ecology and energy consumption index. In this study, the termite mound chamber is biomimicked from a biological point of view. Combining the structural form of termite mound chambers with the structural form of high-rise buildings, the temperature control mechanism and wind circulation performance of bionic termite mound chambers are deeply bionicized through the technical methods of increasing the chamber structure, changing the location of chambers, and increasing the number of chambers in high-rise buildings by using CFD simulation technology. The study maximizes the use of the natural ventilation mode to achieve the optimization of the connectivity and ventilation performance of high-rise buildings and realizes the high-efficiency mode of the chamber structure of high-rise buildings, thereby reducing the energy consumption of high-rise buildings and enhancing the sustainable design of buildings.

The simulation experiment firstly proposes adaptive changes to the CARRC high-rise building model, and on the basis of adding the main chamber structure of the bionic termite mound, three kinds of single-attachment chamber structures and three kinds of double-attachment chamber structures with a total of seven modes, through the CFD simulation results, it can be seen that: (1) The increase in the main chamber structure of the bionic termite mound has a negligible impact on the wind environment around the high-rise building, and at the same time, through the standardized average wind velocity comparison, it is confirmed that increasing the main chamber structure can enhance the wind speed of indoor ventilation of the high-rise building, which is conducive to the comfort of the users. (2) On the basis of the main chamber structure in the high-rise building model, the attached chamber of the bionic termite mound is added to the high-rise building model with a single-attached chamber or double-attached chambers, which has a negligible impact on the wind environment around the high-rise building, the wind speed is greater on the same standard floor near the attached chambers, and the impact is strongly varied by the proximity to the chambers. Relative to the traditional structure of the building without chambers, we increase the setup of the bionic termite mound chamber structure of the high-rise building; its wind speed and wind pressure in the sample sampling floors are higher than the traditional high-rise building without chambers, the maximum difference between the two models of the wind speed is 0.05 m/s, the maximum difference between the wind speed of a single building is 0.14 m/s, and the high-rise building with a chamber can meet the natural ventilation environment. (3) Models with the addition of single- or double-attached chambers are better for promoting the ventilation performance of high-rise buildings than those with the addition of only the main chambers. After adding the single-attached chamber structure of a bionic termite mound, the difference of the sampled wind speed of different floors reaches 0.15 m/s, which is 0.10 m/s more than that of the model with only the main chamber of the high-rise building. In the bionic termite mound chamber high-rise building with a double-attached chamber model, the maximum difference in wind speed of each floor sampling point can reach 0.19 m/s, while the wind pressure maps show a stable wind environment system. (4) In the dual-chamber bionic termite mound high-rise building chamber model, the model of the nine floor sampling points of the maximum wind speed difference value of 6 has the highest value, the single building wind speed difference value has the smallest value of 0.10 m/s, and the building ventilation performance is more efficient and stable. The comparison of the double-attached chamber model for the ventilation performance of high-rise buildings is better than adding a single-attached chamber, and through the data comparison it can be concluded that for the overall ventilation environment optimization of high-rise buildings, the a4 model (set up in the B and C part of the double-attached chamber) is a more efficient model.

Through this study, it can be found that the chamber structure of termite mounds has positive significance for the ventilation optimization of high-rise buildings, which is a solution to the dilemma of high-rise building ventilation. However, due to the limitations of the study, this paper only discusses two wind environment factors, namely, wind speed and wind pressure, for the ventilation performance of high-rise buildings with bionic termite mound chamber structures. Meanwhile, due to the limitation of the building’s morphological structure, the high-rise building model only simulates the wind environment of the attached chamber on the side of the bionic termite mound chamber close to the main chamber, while the attached chambers on the different sides are not discussed in depth. Accurate data tracking the air flow (e.g., the circulation pattern of colored gases with markers) within the termite mound chamber also require further experimental setups and more scientific measurements, which is one of the next directions for us to deepen our research. Meanwhile, the analysis of data samples of other environmental evaluation indexes, such as air temperature, humidity, and air age, is still to be followed by further research.

## Figures and Tables

**Table 1:** Wind speeds and differences at different floors for a high-rise building model without a chamber and a high-rise building model with a chamber.

High-Rise Building Model	Average Value of Wind Speed at Different Floors (m/s)									Monomers Maximum Wind Speed Difference
	3F	8F	11F	15F	19F	22F	26F	29F	33F	
Chamber-free high-rise building model	0.09	0.1	0.11	0.12	0.13	0.14	0.14	0.17	0.18	0.09
High-rise building model with a chamber	0.10	0.12	0.13	0.16	0.18	0.18	0.19	0.23	0.24	0.14
Maximum wind speed difference	0.01	0.02	0.02	0.04	0.05	0.04	0.05	0.06	0.04	0.05

**Table 2:** Wind speeds and differences between single-chamber and double-chamber high-rise building models at different floors.

High-Rise Building Model		Average Value of Wind Speed at Different Floors (m/s)									Monomers Maximum Wind Speed Difference
		3F	8F	11F	15F	19F	22F	26F	29F	33F	
Single chamber	a1	0.20	0.21	0.15	0.16	0.18	0.18	0.19	0.26	0.28	0.08
	a2	0.12	0.14	0.15	0.22	0.26	0.20	0.24	0.27	0.30	0.18
	a3	0.11	0.12	0.15	0.16	0.18	0.18	0.20	0.30	0.34	0.23
Dual chamber	a4	0.21	0.22	0.25	0.28	0.28	0.27	0.29	0.30	0.31	0.10
	a5	0.22	0.24	0.23	0.22	0.21	0.22	0.24	0.27	0.33	0.11
	a6	0.15	0.17	0.17	0.18	0.19	0.24	0.27	0.30	0.34	0.19
Model maximum wind speed difference		0.11	0.10	0.10	0.12	0.10	0.09	0.10	0.04	0.06	0.15

## Data Availability

Data are available upon request.
